# Monitoring drug metabolic pathways through extracellular vesicles in mouse plasma

**DOI:** 10.1093/pnasnexus/pgae023

**Published:** 2024-01-23

**Authors:** Xiaofeng Wu, Menchus Quan, Marco Hadisurya, Jianzhong Hu, Yi-Kai Liu, Yuxin Zhuang, Li Li, Anton B Iliuk, Jun J Yang, Shihuan Kuang, W Andy Tao

**Affiliations:** Department of Chemistry, Purdue University, West Lafayette, IN 47907, USA; Department of Biological Sciences, Purdue University, West Lafayette, IN 47907, USA; Department of Biochemistry, Purdue University, West Lafayette, IN 47907, USA; Department of Pharmaceutical Sciences, St Jude Children's Research Hospital, Memphis, TN 38105, USA; Department of Biochemistry, Purdue University, West Lafayette, IN 47907, USA; Department of Biochemistry, Purdue University, West Lafayette, IN 47907, USA; Tymora Analytical Operations, West Lafayette, IN 47906, USA; Department of Biochemistry, Purdue University, West Lafayette, IN 47907, USA; Tymora Analytical Operations, West Lafayette, IN 47906, USA; Department of Pharmaceutical Sciences, St Jude Children's Research Hospital, Memphis, TN 38105, USA; Department of Oncology, St Jude Children's Research Hospital, Memphis, TN 38105, USA; Department of Biological Sciences, Purdue University, West Lafayette, IN 47907, USA; Department of Animal Sciences, Purdue University, West Lafayette, IN 47907, USA; Purdue Institute for Cancer Research, Purdue University, West Lafayette, IN 47907, USA; Department of Chemistry, Purdue University, West Lafayette, IN 47907, USA; Department of Biochemistry, Purdue University, West Lafayette, IN 47907, USA; Tymora Analytical Operations, West Lafayette, IN 47906, USA; Purdue Institute for Cancer Research, Purdue University, West Lafayette, IN 47907, USA

**Keywords:** drug ADME, extracellular vesicles, proteomics, P450 enzymes, mass spectrometry

## Abstract

The ability to monitor the response of metabolic enzymes to drug exposure in individuals is highly appealing and critical to personalized medicine. Although pharmacogenomics assesses genotypic differences, it does not report changes in metabolic enzyme activities due to environmental factors such as drug interactions. Here, we report a quantitative proteomics strategy to monitor drug metabolic pathways by profiling metabolic enzymes in circulating extracellular vesicles (EVs) upon drug exposure. Mass spectrometry (MS)-based measurement revealed that changes in metabolic enzyme abundance in EVs paralleled those in hepatic cells isolated from liver tissue. Coupling with multiplexed isotopic labeling, we temporally quantified 34 proteins involved in drug absorption, distribution, metabolism, and excretion (ADME) pathways. Out of 44 known ADME proteins in plasma EVs, previously annotated mouse cytochrome P450 3A11 (Cyp3a11), homolog to human CYP3A4, and uridine 5'-diphospho (UDP) glucuronosyltransferase 2A3 (Ugt2a3), increased upon daily rifampicin dosage. Dasatinib, a tyrosine kinase inhibitor to treat leukemia, also elevated Cyp3a11 levels in plasma EVs, but to a lesser extent. Altogether, this study demonstrates that measuring drug enzymes in circulating EVs as an effective surrogate is highly feasible and may transform today's drug discovery and development for personalized medicine.

**Significance Statement**

It remains a challenge to measure the absorption, distribution, metabolism, and excretion (ADME) proteins in biofluids to evaluate drug efficiency. By incorporating the tandem mass tag strategy and efficient isolation of extracellular vesicles (EVs) from murine serum, we achieved accurate quantification of 34 important drug-induced ADME proteins including sixteen P450 enzymes. Noticeably, changes in certain enzyme abundance in plasma EVs were correlated to drug dosage. The strategy allows for the measurement of ADME proteins in EVs as potential biomarkers that may transform the clinical characterization of ADME variability through routine liquid biopsy.

## Introduction

Characterization of drug absorption, distribution, metabolism, and excretion (ADME) activities is critical to assess drug efficacy and toxicity (1, 2). To date, clinical pharmacogenetic evaluation of hepatic cells has been the primary approach to distinguish gene polymorphisms of ADME proteins, especially cytochrome P450 (CYP) enzymes (3–7). However, mere genetic analysis is insufficient to reveal real-time phenotypic ADME performance, for example, clinical metabolic robustness (5, 8, 9). On the other hand, *ex vivo* and in vivo phenotypic studies have focused primarily on ADME enzymes in liver tissues or primary hepatocytes, which lack clinical feasibility through routine monitoring or regular examinations (10–12).

Recently, biofluid extracellular vesicles (EVs) and their cargos have emerged as promising biomarker surrogates for many diseases. With nucleic acids, proteins, and metabolites encapsulated by a double-layer membrane, EVs present “snapshots” of their parental cells (13–17). It is conceivable that ADME proteins in EVs, if detectable and measurable, can be used to evaluate immediate drug response through liquid biopsy, pointing to exciting clinical perspectives of noninvasive sampling and phenotypic drug ADME monitoring for personalized medicine. Previous attempts had to use either in-gel tryptic digestion on P450 enzyme bands or inefficient immunoprecipitation of hepatic EV subpopulations to enrich EV ADME proteins for targeted measurements by pseudo- or normal-multiple reaction monitoring (18–20).

**Fig. 1.**
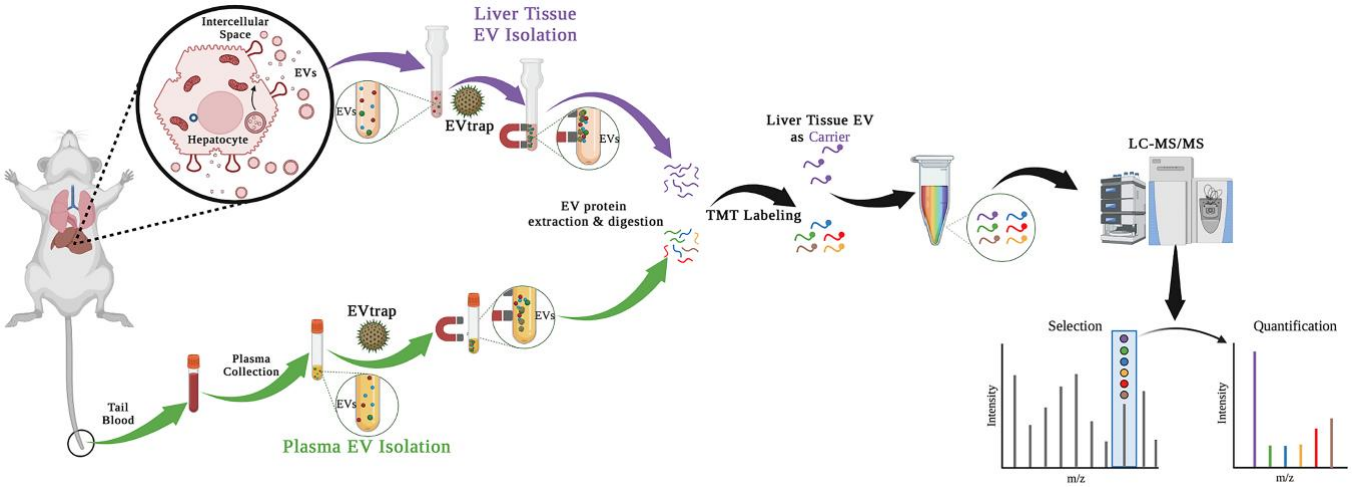
The workflow for plasma EV proteomics aiming to monitor liver ADME status. After sampling blood/plasma from mice of vehicle control or drug treatment, carrier liver tissue, and plasma EVs were isolated by EVtrap, followed by protein extraction, digestion, and TMT labeling for LC–MS/MS analyses.

Here, we integrated an efficient technique based on functionalized magnetic beads, termed EV total recovery and purification (EVtrap), for EV isolation (21, 22) with tandem mass tag (TMT) multiplexed isobaric labeling to achieve sensitive and quantitative measurement of ADME proteins in plasma EVs (Fig. 1). The approach adopts a signal-enhancing strategy in which a large number of relevant proteins, used as carriers, are labeled with one TMT reagent and then mixed with the labeled low-abundance plasma EV samples using other TMT channels, enabling the measurement of ADME proteins in biofluids with enhanced detection sensitivity and sample throughput. Among 44 ADME proteins consistently identified and 34 confidently quantified, Cyp3a11 (a homolog to human CYP3A4), UDP glucuronosyltransferase 2A3 (Ugt2a3) and other ADME proteins in mouse plasma EVs appeared as potential indicators of drug response (23, 24). The study shed light on the feasibility of achieving ADME phenotypic surveillance through clinical liquid biopsy.

## Results

### Measurement of ADME proteins in liver tissue EVs

Before analyzing patient biofluid samples, such as plasma and serum, where EVs can originate from any cell, careful examination of cell line-derived EVs in vitro is essential for clinical EV research (25, 26). However, EV properties may be distorted after—prolonged cell culture, as observed in the numerous mutations that occur during tumor cell proliferation (27, 28). Furthermore, EV studies on cell lines fail to represent the cellular heterogeneity within the tissues of interest, missing information from other tissue cell populations (29, 30). Therefore, analysis of EVs in the interstitial space within a specific tissue (tissue EVs) is a more accurate representation of in vivo tissue status and is crucial to future biofluid EV investigations (31, 32).

**Fig. 2.**
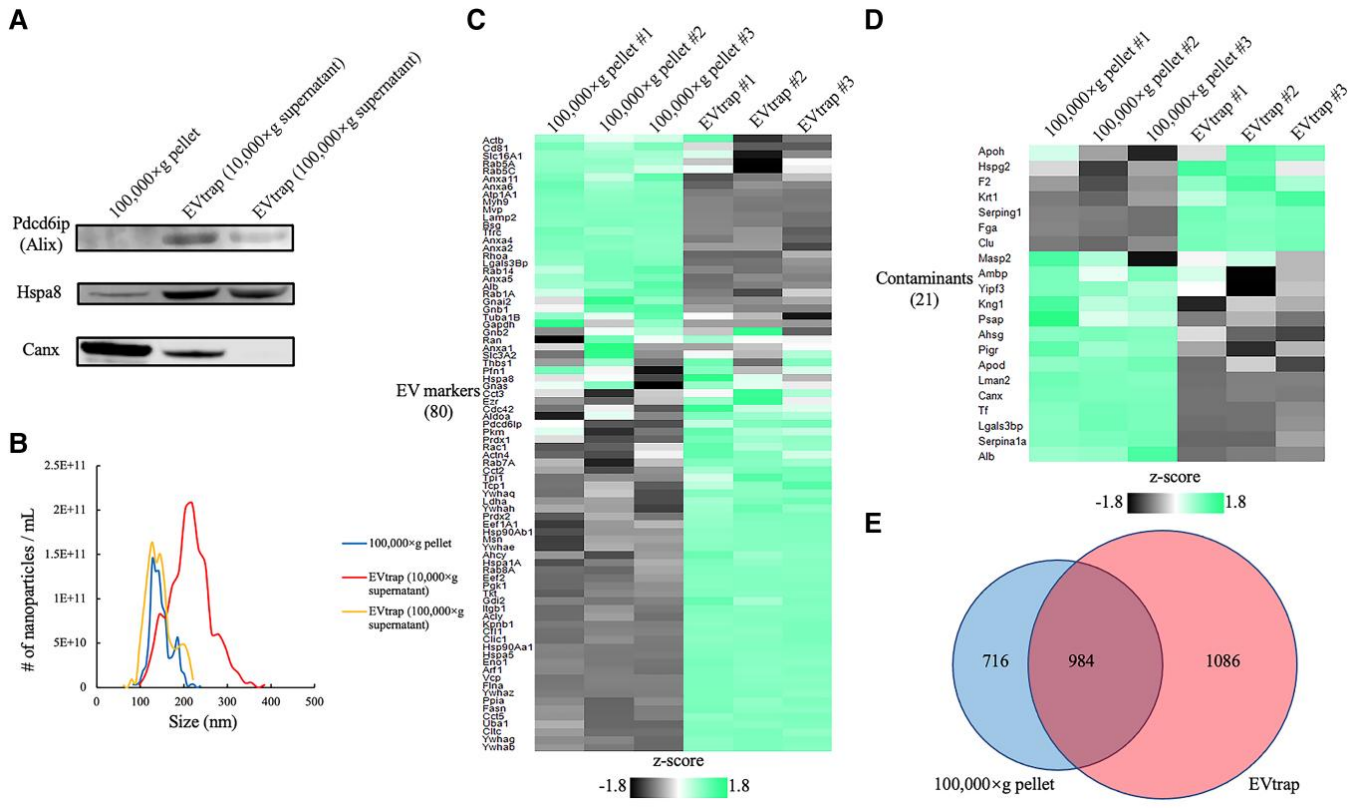
Comparison between ultracentrifugation (100,000 × *g*) and EVtrap for liver tissue EV isolation. Particles were isolated from 0.2 mL supernatant after 10,000 × *g* centrifugation on tissue homogenate. A) Western blotting data showing programed cell death six-interacting protein (Pdcd6ip, also known as Alix) and heat shock protein family A member 8 (Hspa8) as EV markers, as well as Calnexin (Canx) as contaminant protein (negative control). B) Nanoparticle analysis was performed by TRPS on 100,000 × *g* pellets or eluates from EVtrap beads. Relative abundances of representative C) EV markers and D) contaminant proteins by LC–MS/MS analysis with the *z*-score color indicated, respectively. 0.5 μg resulting peptides from each method were loaded and technical triplicates were employed. E) The numbers of identified EV proteins by ultracentrifugation and EVtrap isolation methods. Selected proteins were identified in at least two out of three technical replicates.

Our group recently introduced a novel strategy and material, termed EVtrap, featuring magnetic beads functionalized with hydrophilic and lipophilic molecules to achieve efficient isolation of biofluid EVs through chemical affinity from urine and plasma (21, 22). Here, we expanded the application of EVtrap to isolate EVs in situ from tissue samples (Figs. 1 and S1) (29, 33). EV recovery efficiency by EVtrap was much higher than standard ultracentrifugation, based on immunoblotting results using two representative EV markers and the nanoparticle size distribution by tunable resistive pulse sensing (TRPS; Fig. 2A and B). In addition, the EVtrap eluate contained lower levels of contaminant proteins such as calnexin (Canx; Figs. 2A and S2A). Consistently, when loading an equal amount of digested peptides for Liquid Chromatography–mass spectrometry (LC–MS) analysis, more known EV proteins were identified with a lower amount of contaminants in the EVtrap samples compared to the ultracentrifuge samples (Figs. 2C–E and S2B–D). Moreover, we could still recover more EVs with EVtrap from the traditionally believed “EV-free” supernatant after 100,000 × *g* ultracentrifugation (Fig. S3). These results indicate that EVtrap is a more specific and efficient method for EV isolation, and represents an ideal tool for detecting ADME proteins in EVs.

**Fig. 3.**
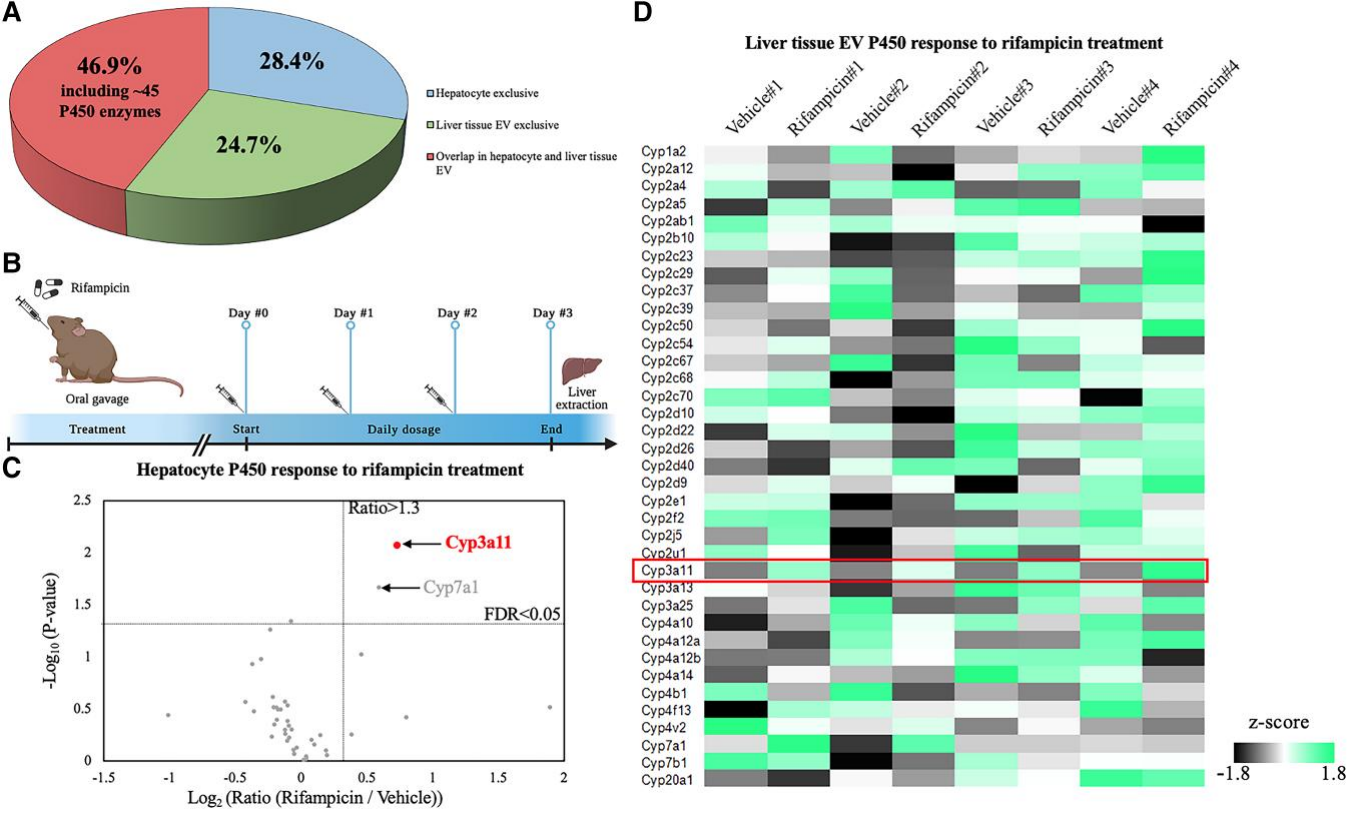
Liver tissue EVs serving as “snapshots” of hepatocytes. A) Identified protein distribution in hepatocytes and liver tissue EVs by LC–MS/MS. B) Three-day vehicle or rifampicin treatment on four pairs of mouse littermates resulting in the regulation of P450 enzymes in C) hepatocytes and D) liver tissue EVs, respectively. All P450 enzymes were quantified by label-free LC–MS/MS analysis. Among all four pairs, the highlighted Cyp3a11 was identified as the promising P450 biomarker for rifampicin stimulation, in both hepatocytes and liver tissue EVs.

Next, cells and EVs were isolated from mouse tissues. Although liver tissue contains many different cell types, the hepatocytes account for up to 80% of the overall liver volume. Thus, the majority of whole liver tissue EV populations should be hepatic EVs (34, 35). Using biological mouse liver triplicates, 4,290 proteins on average were identified in the liver tissue without prefractionations, and 4,076 proteins were detected in liver tissue EVs (Fig. S4A). In the total identified protein list, 28.4% was exclusively identified in hepatocytes, 24.7% exclusively in liver tissue EVs, and 46.9% were shared by both (Figs. 3A and S4B). This observation indicates that many hepatocyte proteins can be detected in EVs. As we did not use extensive prefractions on whole cell extracts, only partial overlap between EV proteins and cellular proteins was expected. It is also possible that some proteins were deliberately packaged into EVs and secreted with EVs, leaving only undetectable amounts inside the cell. Through gene ontology (GO) analysis, many EV proteins were significantly enriched in processes like vesicle-involved transport or trafficking, and components like exosome formation and cargo enveloping, indicating high isolation specificity by EVtrap (Fig. S5D). Most noticeably, the “overlapping” proteins in both hepatocytes and EVs included 45 P450 family enzymes on average (Fig. S4B), these being particularly relevant in a variety of metabolic and catabolic processes (Fig. S5B and C).

### ADME protein dynamics in liver tissue EVs reflect those in hepatocytes

We first examined the level of ADME proteins in liver EVs in response to treatment with an antibiotic drug rifampicin. Rifampicin or vehicle control was administrated via three daily IP injections to four pairs of sex-matched littermate mice with the dosage proportional to individual body weight (Fig. 3B). Mice were sacrificed 24 h after the last dose for liver tissue extraction, followed by hepatocyte harvest and EV isolation. Cells and EVs were lysed to extract proteins to generate peptides followed by label-free quantitative LC–MS/MS analysis (Fig. S1). As expected, Cyp3a11, the mouse homolog of human CYP3A4, was found to be consistently increased in all four rifampicin-treated mice in both hepatocytes and tissue EVs, by 66 and 89% increase on average, respectively (Figs. 3C and D and S6). The MS results were consistent with the *Cyp3a11* transcriptional induction at the mRNA level reported in other studies (36, 37), underscoring that ADME enzymes in liver tissue EVs reflected the hepatocyte drug metabolism.

### Monitoring ADME protein changes in plasma EVs in response to drug exposure

Blood enters the liver via the hepatic artery and portal vein, and leaves through the hepatic veins (38). Liver tissue EVs that are generated by resident cells then enter systemic circulation through the same path. This fact substantiates the feasibility of clinically monitoring the drug ADME by liquid biopsy via circulating EVs.

**Fig. 4.**
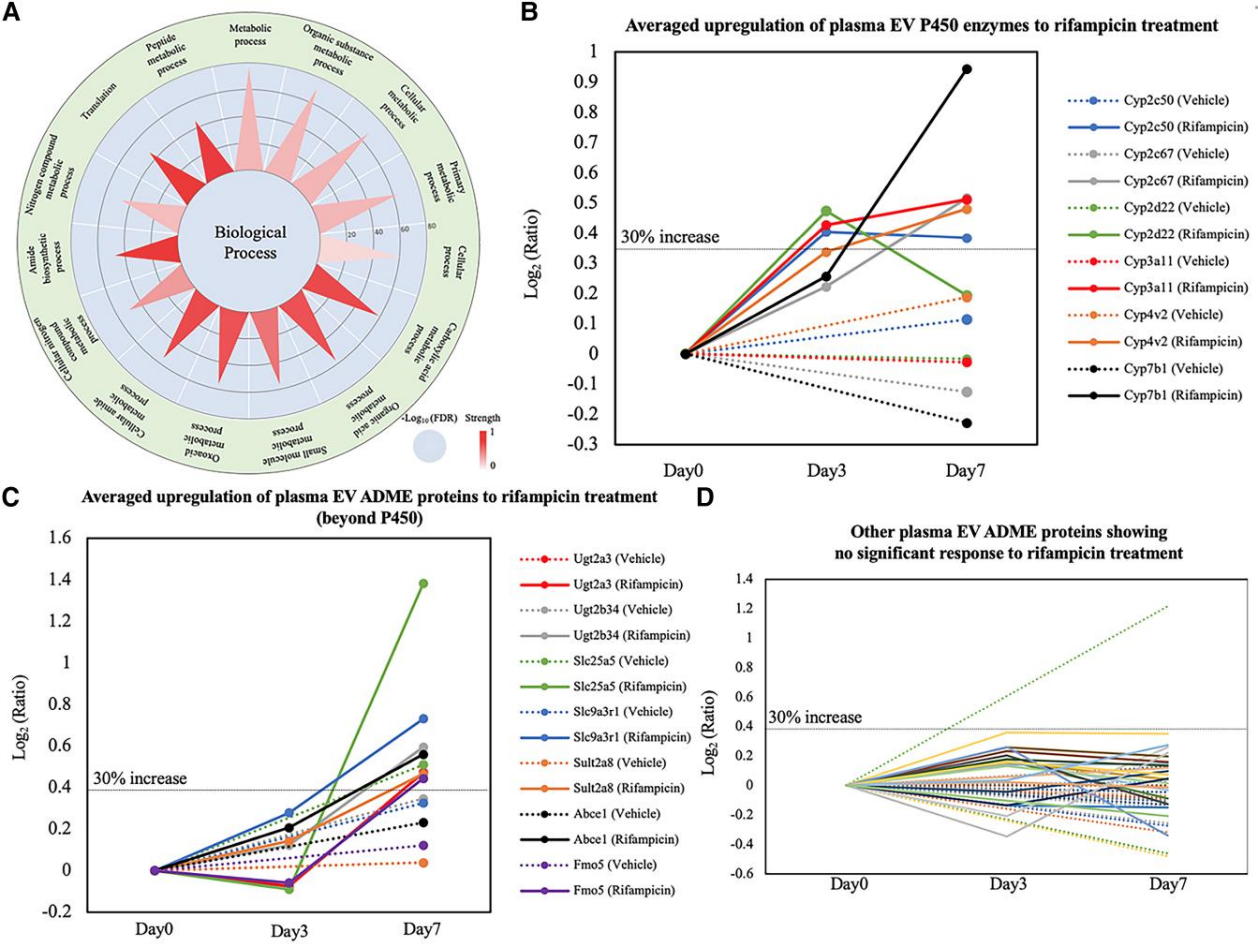
Plasma EV proteomic identification and quantitation by TMT carrier strategy to describe the regulation of ADME proteins from vehicle controls (*n* = 3) and rifampicin treatment trials (*n* = 3). A) Classification of identified plasma EV proteins in TMT carrier experiments based on biological process, emphasizing that multiple liver-focused metabolic processes were enriched with liver tissue EV as TMT carrier. As our focus, 13 ADME proteins showed significant upregulation average, including B) six proteins within P450 family and C) seven proteins beyond P450. Meanwhile, the other 21 ADME proteins showed rifampicin-causing upregulation <30% D), with “vehicle” trials in dash lines and “rifampicin” trials in solid lines.

After daily dosage of vehicle or rifampicin for a week, 10–20 μL tail blood was drawn in each of three pairs of mouse littermates (Fig. S7). Our initial analyses based on label-free quantitation were unsatisfactory due to the weak intensity of ADME proteins in plasma EVs. Therefore, in the following LC–MS/MS analyses, we incorporated multiplexed isobaric labeling based on TMT reagents to improve the identification and quantitation of ADME proteins in plasma EVs. The liver tissue EV sample with relatively higher levels of ADME proteins served as the carrier and was labeled by one TMT channel, it was then mixed with plasma EV samples labeled by other TMT channels. In total, 1,082 proteins were identified from three pairs of mouse samples, among which many proteins were involved in various metabolic activities (Figs. 4A and S8). Forty-four ADME proteins were consistently identified and 34 were confidently quantified. According to averaged variation ratios of ADME proteins upon rifampicin treatment, 13 out of 34 displayed significant upregulation (>30%) on either day 3 or day 7, leaving the 21 remaining ADME proteins expressing stably for the duration of the treatment period (Fig. 4B–D).

**Fig. 5.**
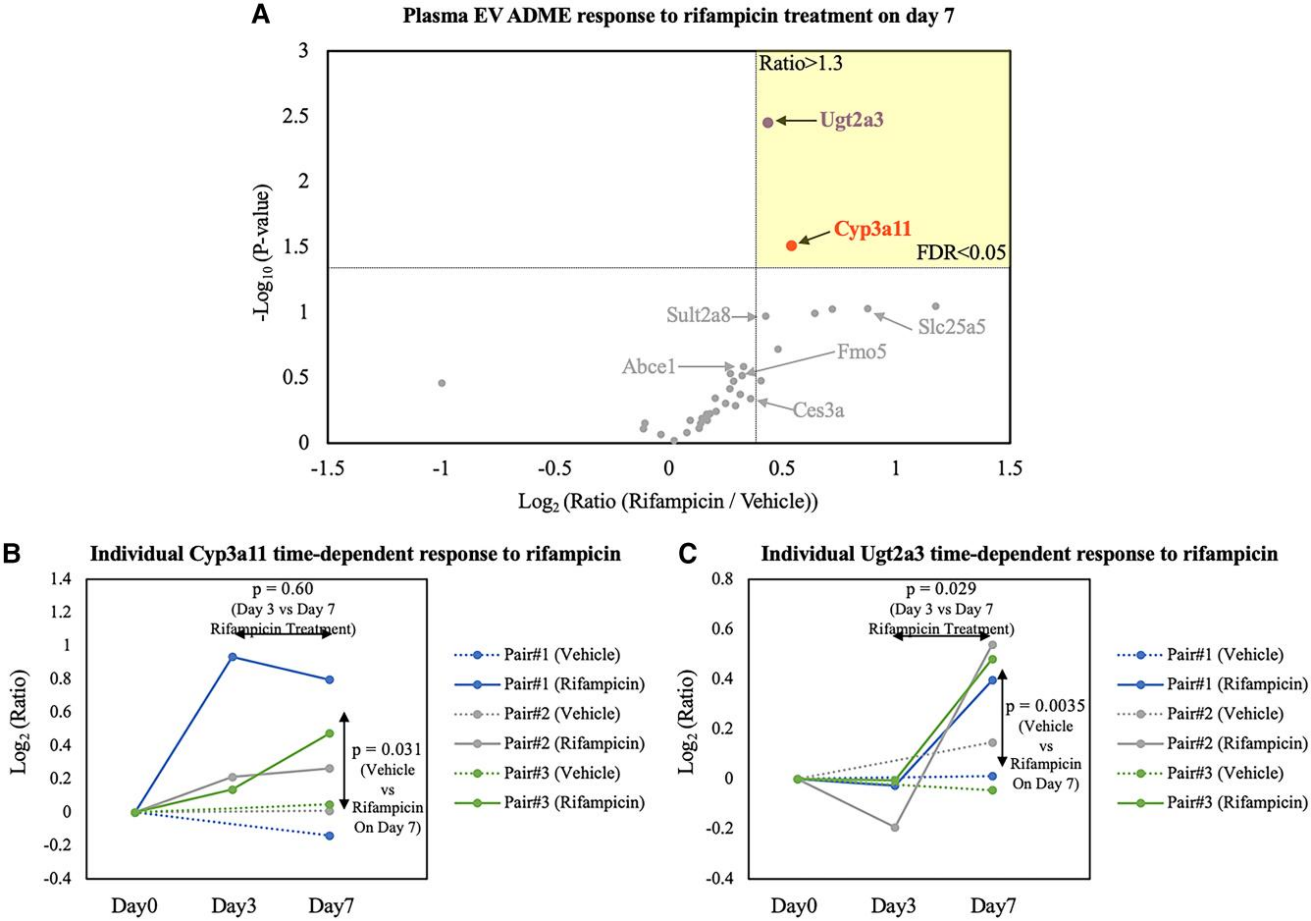
Drug-stimulation time-dependent response in plasma EVs. A) Volcano plot highlighting comparative ADME response to rifampicin treatment on day 7. Among various ADME proteins, Cyp3a11 and Ugt2a3 in plasma EVs emerged with the most consistent and significant induction (FDR < 0.05), from three pairs of biological replicates. Response of B) Cyp3a11 and C) Ugt2a3, respectively, to rifampicin along 7-day treatment was illustrated individually and *P*-values of treatment response were plotted at designated positions.

When the vehicle baseline and regulation consistency were examined, Cyp3a11 was the only reliable P450 biomarker among the six responsive P450 enzymes (Figs. 5A and B). An average increase of 35% was shown in Cyp3a11 after 3 days of rifampicin treatment and a >40% increase after the last dose on day 7. This result may be explained by the fact that rifampicin induces not only Cyp3a11 expression but also other enzymes that metabolize the drug, hindering its effects. This would be consistent with our observation that Cyp3a11 abundances came near a plateau or even dropped a bit after 7-day treatment (Fig. 5B). As shown in our liver EV experiments, Cyp3a11 is a reliable indicator of rifampicin pharmacodynamics in hepatocytes. With our plasma EV data, circulating Cyp3a11 was observed to reflect a real-time drug response. Additionally, due to the high promiscuity of P450 metabolism, rifampicin is metabolized by human CYP2C8, 2C9, and 2C19 as well, and causes their induction (39). From the search results by Basic Local Alignment Search Tool (BLAST), Cyp2c50 shared ∼70% sequence similarity with human CYP2C8, 2C9, and 2C19. Therefore, a significant Cyp2c50 increase in our data was expected (Fig. 4B) (40). The fact that Cyp3a11 serves as the primary enzyme in rifampicin metabolism diminishes the role of other P450 enzymes in this context, possibly explaining the low consistency of Cyp2c50 induction.

Outside of the P450 family, UDP glucuronosyltransferase 2A3 (Ugt2a3) expression was observed to be responsive to rifampicin (Figs. 4C and 5C), which is consistent with results reported in a previous in vitro study (41). ADME proteins like Ugt2a3 and solute carrier family 25 member 5 (Slc25a5) displayed minimal response after short-term drug exposure (3 days), but were greatly induced on final-day detection according to plasma EV data (Figs. 4C, 5C, and S9A) (42). The data were consistent with the outcomes from hepatocytes and liver tissue EVs in which the level of Ugt2a3 and Slc25a5 barely changed after 3 days of rifampicin treatment (Fig. S10). Other ADME proteins such as sulfotransferase (Sult2a8), carboxylesterases (Ces3a), adenosine triphosphate (ATP)-binding cassette (Abce1), and flavin-containing monooxygenase (Fmo5), had less significant responses to rifampicin treatment (Fig. S9B–E). Still, many trends in our data followed previous literature reports. For example, the human homolog to mouse Sult2a8, SULT2A1, was detected to increase at the mRNA level in over 50% of patient-donated primary hepatocyte models upon rifampicin treatment (43). In other ex vivo rifampicin-stimulation experiments regarding Fmo5, increased expression levels were found in both human and mouse hepatocytes (44, 45).

Meanwhile, we also examined the ADME proteins in plasma EVs relevant to tyrosine kinase inhibitor dasatinib (Sprycel) (46, 47). Known as a milder CYP3A4 inducer and acting under a time-dependent inhibition (TDI) mechanism, dasatinib caused a ∼20% increase of Cyp3a11 on day 3 but the Cyp3a11 level dropped even with continuous treatment of dasatinib (Fig. S11A). Elevation of Ces3a (24%) and Abce1 (40%) was also observed on day 3 (Figs. S11B and C). Similar to the pattern of Cyp3a11 regulation after continued treatment of dasatinib, abundances of both Ces3a and Abce1 returned close to baseline on day 7. Although rifampicin is a commonly used model drug in ADME studies, dasatinib has been widely marketed and deemed safe to use for most patients, benefitting from its less dramatic pharmacodynamic effects.

## Discussion

Current ADME detection for drug discovery at clinical phases is primarily based on drug degradation or metabolite formation but misses ADME protein information for pharmacodynamic portfolios. Also, in clinical scenarios, real-time ADME protein detection still lacks effective analytical methods for precise drug efficacy and toxicity evaluation for each patient. Here, we turned our attention to biofluid EVs as surrogates of ADME proteins. Using mouse tail blood as one example of convenient sampling, we presented a robust analytical pipeline that included EV isolation by magnetic bead-based EVtrap, protein processing, and TMT-LC–MS/MS analyses. Using liver tissue EVs as the carrier, a wide range of ADME proteins were detected in plasma EVs and quantified. As a proof-of-principle study, we successfully identified well-verified Cyp3a11 and Ugt2a3 as reliable indicators of metabolic response in vivo to rifampicin treatment. Additional data using dasatinib treatment demonstrated the possible comparison of ADME performance among various drugs. The strategy demonstrated the feasibility of discovering ADME markers for specific drug monitoring by liquid biopsy. With ADME proteins being detected in biofluids, it is feasible to carry out efficacy measurements during clinical phases. The study also presents the possibility of monitoring individual's drug metabolism performance, a critical part of personalized medicine.

Clearly, this promising strategy is at the early stage of development before its clinical applications. While we carried out a time course with the drug treatment using mouse models to investigate ADME dynamics in EVs, a more detailed time-course study is needed to find the optimum time window for EV sample collection from plasma. It is also essential to quantify the run down time of the ADME protein concentrations in EVs in response to drug treatment. The sensitivity and reproducibity of the approach need to be examined with a larger sample size, and ultimately with human plasma samples, by measuring key ADME proteins. The success of the method depends heavily on the efficient and specific isolation of circulating EVs from plasma, while the isolation of EVs remains a challenge, in particular from plasma samples. We demonstrated the EVtrap technique, while not perfect, allowed for the generation of relatively clean liver tissue EVs as suitable TMT carriers. EVtrap also achieved more efficient EV isolation from limited plasma volumes, as low as 10–20 μL, compared to other EV isolation methods such as ultracentrifugation, to significantly improve the assay sensitivity. Thus, in the isolation of tissue EVs and plasma, or other biofluid EVs, we incorporated EVtrap in a robust analytical pipeline. EVs are heterogeneous in their nature, and plasma EVs may come from different organs and different cell types. Because the current strategy does not allow us to enrich liver-specific EVs, the biggest challenge is its sensitivity and high background. The TMT carrier method has been leveraged to increase the sensitivity from trace amounts of starting samples, for example, in single-cell proteomics (48, 49). In our experiments, the strategy, in combination with the EVtrap isolation, was adopted to purposely identify and quantify ADME proteins in plasma EVs. However, the innate drawbacks of TMT labeling are unavoidable, especially with confounding peptides within the MS1 isolation window leading to quantitation distortion at the MS2 stage. We attempted to incorporate peptide fractionation to mitigate the interference effect, but for high-throughput discovery purposes, this was an inefficient solution, not applicable in clinical settings. This highlights the need for alternative solutions such as targeted proteomic quantitation (e.g. multireaction monitoring, MRM) and the development of liver-specific EV isolation.

In plasma EVs from mouse models, Cyp3a11 and Ugt2a3 were discovered as two candidate markers induced by rifampicin, as we expected from hepatocyte culture results and previous reports. However, multiple ADME protein expression patterns did not match those in hepatocytes and liver tissue EVs. There are several reasons for the translational barrier between an in vitro liver model and circulating biofluids. First, although the liver is where the majority of drug metabolism happens, other organs such as the intestine and kidney are also engaged in drug metabolism. Second, gastrointestinal absorbability and urine or bile excretion rates also greatly influence metabolic response, further adding variations and uncertainties to metabolizing enzyme responses. Third, the rationale of ADME protein secretion into EVs from different organs is still unclear, which possibly distorts the liver-specific EV features. Our most intriguing data were obtained with dasatinib. Dasatinib underwent the TDI mechanism, meaning it irreversibly bound and deactivated Cyp3a11 and triggered the resynthesis of enzymes to compensate. In our dasatinib treatment experiment, it was sensible to monitor detectable changes in plasma EVs within a shorter period of dosage due to this fact. This also highlights further studies to investigate how drug-bound enzymes differ in EV packaging. Altogether, this study demonstrated a promising strategy to in vivo monitor ADMEs in response to drug treatment through plasma EVs, and further implementation will require stringent validation and streamlined bioanalytical pipelines for robust clinical use.

## Materials and methods

### Animals

All animal procedures were performed as per protocol #1112000440 approved by the Purdue University Institutional Animal Care and Use Committee in adherence to the national guidelines. Male and female C57BL/6J mice were used (aged 2–3 months). All experiments used age-matched, litter-matched, and sex-matched controls. Mice were housed and maintained in the animal facility with free access to standard rodent chow and water.

### Drug preparation and administration

Rifampicin (Sigma-Aldrich, R3501) was first dissolved in methanol at a concentration of 25 mg/mL and diluted in distilled water. A vacuum centrifuge was then used to remove methanol and reach the desired concentration of 3.0 mg/mL. Vehicle control (distilled water and methanol subjected to vacuum centrifugation) or rifampicin trials were administered by oral gavage at a dosage of 10 mg/kg mouse body weight. Treatments were given every 24 h for 7 days in a row. Dasatinib (LC Laboratories, D-3307) was dissolved in 80 mM citric acid and daily administrated by oral gavage to 20 mg/kg NOD scid gamma (NSG) mice, with the corresponding vehicle at the same volume of 80 mM citric acid.

### Mouse liver tissue sample extraction and process

Whole livers were isolated from mice following the treatment period of designated time lengths. Livers were then rinsed with 1× phosphate-buffered saline (PBS, homemade) and minced into small pieces (<1 mm in diameter) with scissors. Liver samples were digested in 1.25 mg/mL Collagenase type I (Worthington Bio, LS004197) in serum-free Dulbecco's modified eagle medium (DMEM, Sigma-Aldrich, D5796) at 37 °C for 1 h with occasional end-to-end inversions. Digestion was then terminated with an equal volume of fresh serum-free DMEM and filtered through 70 µm nylon cell strainers to discard undigested tissue and large debris. Keeping all the following steps processed at 4 °C, the resulting supernatant was sequentially centrifuged at 50 × *g* for 10 min to get cells and supernatant, 300 × *g* on supernatant for 10 min to remove other cells, 2,000 × *g* for 20 min to remove dead cells and final 10,000 × *g* for 70 min to remove cell debris and large particles. The 10,000 × *g* supernatant samples were ready for liver tissue EV isolation. Additionally, 50 × *g* spun cells were further purified to obtain hepatocytes, by using 1× red blood cell lysis buffer (Invitrogen, 00-4333) according to the manufacturer's instructions.

### Mouse tail blood and plasma collection

Tail blood used for plasma analysis was collected on day 0 (before treatment), day 3, and day 7, respectively. Blood samples were collected and dispensed into ethylenediaminetetraacetic acid (EDTA) collection tubes and mixed gently to ensure exposure to EDTA-coated walls. Plasma was separated by centrifugation at 1,100 × *g* for 10 min at room temperature. The clear top layer was transferred to another tube and then centrifuged at 1,500 × *g* for 15 min followed by 2,500 × *g* for 10 min, to completely remove platelets, apoptotic bodies, and other large particles and aggregates. The 2,500 × *g* supernatant samples were ready for plasma EV isolation.

### Liver tissue EV isolation by ultracentrifugation

As a control method for EV isolation, prepared liver tissue 10,000 × *g* supernatant samples were centrifuged at 100,000 × *g* for 70 min at 4 °C (Optima MAX-XP Ultracentrifuge, Beckman Coulter). Pellets were resuspended with 1× PBS and spun down at 100,000 × *g* again at the same temperature and time (33). After removing PBS, the pellets were considered ultracentrifuged EVs.

### EV isolation by EVtrap

EVtrap kits, including EVtrap beads as a suspension in water, were provided by Tymora Analytical Operations and were used as described before (21, 22, 50). In brief, the liver tissue supernatant and prepared plasma samples were conditioned by adding the certain volume of respective loading buffers, and the EVtrap beads were added according to the manufacturer's instructions. Samples were incubated by end-over-end rotation for 30 min. After supernatant removal using a magnetic separator rack, the beads were washed with washing buffer followed by 1× PBS. The EVs were eluted by a 10-min incubation with 200 mM triethylamine (TEA, Millipore-Sigma), and the EV samples were fully dried in a vacuum centrifuge.

### Western blotting

Starting from 200 µL liver tissue 10,000 × *g* resulting supernatant, purified EV samples by ultracentrifugation or EVtrap were boiled in 20 µL lithium dodecyl sulfate (LDS) sample buffer with 20 mM Dl-dithiothreitol (DTT, Sigma-Aldrich). Aliquots of each sample were loaded at the equivalent volumes and were separated on a sodium dodecyl sulfate–polyacrylamide gel electrophoresis (SDS–PAGE) gel (NuPAGE 4–12% Bis–Tris Gel, Thermo Fisher Scientific). The proteins were transferred onto a low-fluorescence polyvinylidene fluoride (PVDF) membrane (Millipore-Sigma), and the membrane was blocked with 1% bovine serum albumin (BSA) in tris-buffered saline with 0.1% Tween 20 detergent (TBST) buffer for 1 h. The membranes were then incubated overnight in 1% BSA in TBST, with mouse anti-Alix (Cell Signaling Technology, 3A9, mAb #2171), rabbit anti-Hspa8 (Cell Signaling Technology, D12F2), and rabbit anti-Calnexin (Novus, NB100-1965) based on manufacturer's instructions. The secondary antibodies visualizing the binding of the primary antibody were goat anti-Mouse Alexa Fluor 680 nm and goat anti-Rabbit Alexa Fluor 800 nm (Thermo Fisher Scientific), incubated in the dark for 1 h in 1% BSA in TBST. The membrane was scanned by Odyssey near-infrared scanner (Licor) at 700 and 800 nm wavelength channels for parallel signal detection and quantitation.

### Gel silver staining

The samples were prepared and separated on a gel as described in western blotting. After electrophoresis, SDS–PAGE gel was washed in dH_2_O for 30 min and then fixed with 10% acetic acid and 50% ethanol solution for 1 h. The fixed gel was rinsed once with 50% ethanol for 5 min, followed by sensitizing with 0.02% sodium thiosulfate for 2 min. After a 2-min wash step with dH_2_O, the gel was incubated for 20 min in 0.1% AgNO_3_ staining solution. The gel was washed twice with dH_2_O (1 min each) and then developed with a developing solution (2% sodium carbonate/0.015% formaldehyde/0.0008% sodium thiosulfate). The development was stopped by incubating in 1% acetic acid for 5 min and the final image was scanned.

### Tunable resistive pulse sensing

Liver tissue EVs isolated by ultracentrifugation or EVtrap were resuspended in a 1.25-time volume of Measurement Electrolyte solution (volume compared to the starting 10,000 × *g* supernatant). Upon calibration with 100 and 200 nm standard nanoparticles, TRPS measurements were carried out by Izon using qNano Gold instrument (Izon Science) according to the manufacturer's instructions.

### Peptide preparation

The isolated and dried hepatocyte or EV samples were lysed to extract proteins using the phase-transfer surfactant aided procedure (51). First, EVs were solubilized in the lysis solution containing 12 mM sodium deoxycholate, 12 mM sodium lauroyl sarcosinate, 10 mM tris(2-carboxyethyl)phosphine, 40 mM chloroacetamide in 100 mM triethylammonium bicarbonate (TEAB) by incubating for 10 min at 95 °C. The samples were diluted 5-fold with 50 mM TEAB and digested with Lys-C (Wako) at 1:100 (wt/wt) enzyme-to-protein ratio for 3 h at 37 °C. Trypsin (Sigma-Aldrich) was added to a final 1:50 (wt/wt) enzyme-to-protein ratio for overnight digestion at 37 °C. The samples were acidified with trifluoroacetic acid (TFA) to a final concentration of 1% TFA. Ethyl acetate solution was added at a 1:1 ratio to the samples. The mixture was vortexed for 2 min and then centrifuged at 20,000 × *g* for 3 min to separate aqueous and organic phases. The organic phase (top layer) was removed, and the aqueous phase was collected and dried in a vacuum centrifuge, and desalted using top-tip C18 tips (Glygen, part no. TT2C18.96), according to the manufacturer's instructions. Peptide amounts of each sample were measured by Pierce quantitative colorimetric peptide assay (Thermo Fisher Scientific, 23275) according to the manufacturer's instructions. For label-free LC–MS/MS analysis, 0.5 μg of the peptide from each sample was aliquoted and dried in a vacuum centrifuge; for TMT reagent labeling, 10 μg of the peptide from each sample was aliquoted and dried in a vacuum centrifuge.

### TMT labeling

Six-plex or 10-plex TMT reagents (Thermo Fisher Scientific, 90061, lot# WE322738 and 338781) were dissolved in anhydrous acetonitrile (ACN, Thermo Fisher Scientific, 4340863), according to the manufacturer's instructions. Several liver tissue EV peptide samples were pooled to form 10 μg carrier peptides, and they were always applied at the channel of reporter ion at *m/z* 126. The other channels were occupied by 10 μg individual plasma EV peptides. Desalted and dried samples were resuspended in 25 μL 50 mM TEAB, and corresponding 50 μg of TMT reagents were added into each sample, followed by 1 h incubation at 25 °C. The reactions were quenched by hydroxylamine (Sigma-Aldrich, 438227) for 15 min at 25 °C, and six samples were combined unbiasedly. Pooled TMT-labeled peptides were dried in a vacuum centrifuge.

### Off-line fractionation

Basic pH stage-tip fractionation was performed on top-tip C18 tips (Glygen, part no. TT2C18.96). Peptide samples were resuspended in 200 μL, 200 mM ammonium formate (HCOONH_4_, pH 10.0), and loaded onto the tips. Peptides were sequentially eluted into eight fractions with increasing ACN percentage buffers (4, 7, 10, 13, 16, 19, 22, and 80%, vol/vol). All fractions were dried in a vacuum centrifuge.

### LC–MS/MS analysis

Each 0.5 μg dried peptide sample was dissolved in 10.0 μL of 0.05% TFA with 3% (vol/vol) acetonitrile and injected into an Ultimate 3000 nano UHPLC system (Thermo Fisher Scientific). Peptides were captured on a 2-cm Acclaim PepMap trap column and separated on a heated 50-cm Acclaim PepMap column (Thermo Fisher Scientific) containing C18 resin. The mobile phase buffer consisted of 0.1% formic acid in ultrapure water (buffer A) with an eluting buffer of 0.1% formic acid in 80% (vol/vol) acetonitrile (buffer B) run with a linear 70- or 150-min gradient of 6–30% buffer B at a flow rate of 300 nL/min. The UHPLC was coupled online with a Q-Exactive HF-X mass spectrometer (Thermo Fisher Scientific). The mass spectrometer was operated in the data-dependent mode, in which a full-scan MS (from *m/z* 375 to 1,500 with a resolution of 60,000) was followed by MS/MS of the 15 most intense ions (30,000 resolution; normalized collision energy—28%; automatic gain control target [AGC]—2E4, maximum injection time—200 ms; isolation window −1.6 *m/z*; 60 s exclusion). In TMT-labeled LC–MS/MS analysis, with all LC parameters and most of MS parameters remaining the same, MS/MS mode was switched to pick the 10 most intense ions (normalized collision energy—32%; AGC—2E5, maximum injection time—100 ms; isolation window—0.7 *m/z*).

### LC–MS/MS data processing

The raw files were searched directly against the human Swiss-Prot database updated on 2021 June 20, with no redundant entries, using Byonic (Protein Metrics) and Sequest search engines loaded into Proteome Discoverer 2.3.0.523 software (Thermo Fisher Scientific). MS1 precursor mass tolerance was set at 10 ppm, and MS2 tolerance was set at 20 ppm. Search criteria included a static carbamidomethylation of cysteines (+57.0214 Da), variable modifications of oxidation (+15.9949 Da) on methionine residues, and acetylation (+42.011 Da) at the N terminus of proteins. The search was performed with full trypsin/P digestion and allowed a maximum of two missed cleavages on the peptides analyzed from the sequence database. The false-discovery rates of proteins and peptides were set at 0.01. For TMT-labeled analysis, additional modification of TMT (+229.163 Da) was applied: static on lysine and variable at N terminus of peptides. All protein and peptide identifications were grouped, and any redundant entries were removed. Only unique peptides and unique master proteins were reported. All mass spectrometric data have been deposited to the ProteomeXchange Consortium via the jPOST partner repository with the dataset identifier PXD036086 (52).

### LC–MS/MS quantitative analysis

Label-free data were quantified using the label-free quantitation node of Precursor Ions Quantifier through the Proteome Discoverer v2.3.0.523 (Thermo Fisher Scientific) with normalization of total peptide amount. The intensities of peptides were extracted with initial precursor mass tolerance set at 10 ppm, minimum number of isotope peaks as 2, maximum retention time difference (ΔRT) of isotope pattern multiplets—0.2 min, peptide-spectrum match (PSM) confidence false discovery rate (FDR) of 0.01, with hypothesis test of ANOVA, maximum RT shift of 5 min, pairwise ratio-based ratio calculation, and 100 as the maximum allowed fold change. Instead, TMT-labeled data were quantified using the TMT quantitation node of the Reporter Ions Quantifier. With a hypothesis test of ANOVA, each reporter ion of TMT6plex or TMT10plex was quantified with intensities without a specific normalization strategy.

### Bioinformatic analysis

Only unique peptides with at least five amino acids but without missed cleavage, proline interference, and methionine oxidation, were utilized for accurate quantitation and ratio comparison. Hepatocyte proteins were normalized by total peptide amount, instead, liver tissue EV proteins were normalized by the reliable EV marker Hspa8. All clinical sample data were analyzed using the Perseus software (version 2.0.3.1) (53). The intensities of proteins were extracted from Proteome Discoverer search results, and the missing values of intensities were replaced by a normal distribution with a downshift of 1.8 SDs and a width of 0.3 SDs. In volcano plots, the significantly changed proteins in any sample were identified by the *P*-value and change fold: the vertical dotted line represents the difference of the ratio of 1.3 (log_2_ [ratio] = 0.379), which is the strict criteria for the fold change threshold in our study; the horizontal dotted line represents the *P*-value of 0.05 (−log_10_ [0.05] = 1.30), which is significant based on a two-sample t test with a permutation-based FDR cutoff 0.05 for every two datasets. Specifically calculated *P*-values were performed by either paired or unpaired two-sample student t test and confirmed by the Shapiro–Wilk normality test and *F* test to compare two variances. Linear box-and-whiskers plots were focused on the Pdcd6ip (Alix), Hspa8, and Canx, differentiating the groups with default interquartile range and finished in Microsoft Excel. Heatmaps were based on a small set of proteome containing EV markers (provided by ExoCarta), contaminants, or P450 enzymes, additionally, *z*-score normalization was performed to eliminate the dynamic range effects among different proteins (54–56). Enriched pathway, function, and cellular component information were discovered through the online software GOrilla GO tool or STRING network analysis with public accessibility (57, 58).

## Supplementary Material

pgae023_Supplementary_Data

## Data Availability

All data collected as a part of this study (MSMS raw files and database search files) have been deposited to the ProteomeXchange Consortium via the jPOST partner repository with the dataset identifier PXD036086.
