# Supplementary material for: Monitoring drug metabolic pathways through extracellular vesicles in mouse plasma
Source: PNAS Nexus. 2024 Jan 23;3(2):pgae023. doi: 10.1093/pnasnexus/pgae023 (PMC10833468; doi:10.1093/pnasnexus/pgae023)
Supplement: pgae023_Supplementary_Data [file pgae023_supplementary_data.zip › PNASNEXUS-PNASNEXUS-2023-00930R-s02.pdf]

Supplementary Information for

Monitoring drug metabolism pathways through extracellular vesicles in mouse plasma

Xiaofeng Wu, Menchus Quan, Marco Hadisurya, Jianzhong Hu, Yi-Kai Liu, Yuxin Zhuang, Li Li, Jun J. Yang, Anton B. Iliuk, Shihuan Kuang, and W. Andy Tao

W. Andy Tao

Email: watao@purdue.edu

This PDF file includes:

Figure S1 to S11

Legends for Tables S1 to S12

Other supplementary materials for this manuscript include the following:

Tables S1 to S12

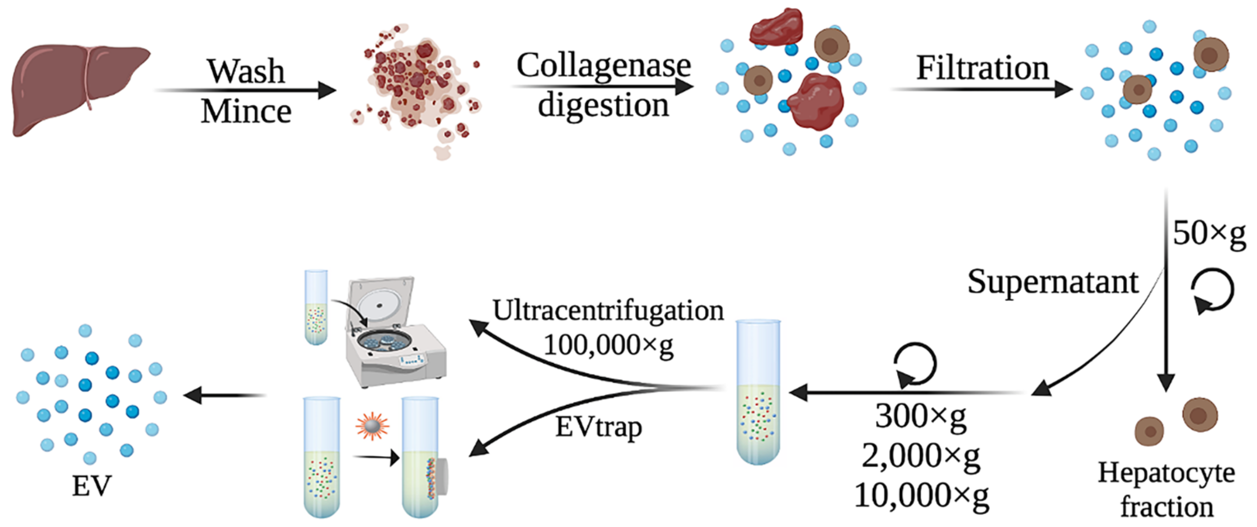

Fig. S1. The workflow of liver tissue extraction and EV isolation. Mouse liver was extracted, washed, minced and digested by collagenase. After filtration to remove large bulks, sequential centrifugation steps were incorporated to isolate hepatocytes and discard other cells, dead cells and cell debris. Starting from 10,000×g supernatant, EVs were isolated by ultracentrifugation (100,000×g) or EVtrap.

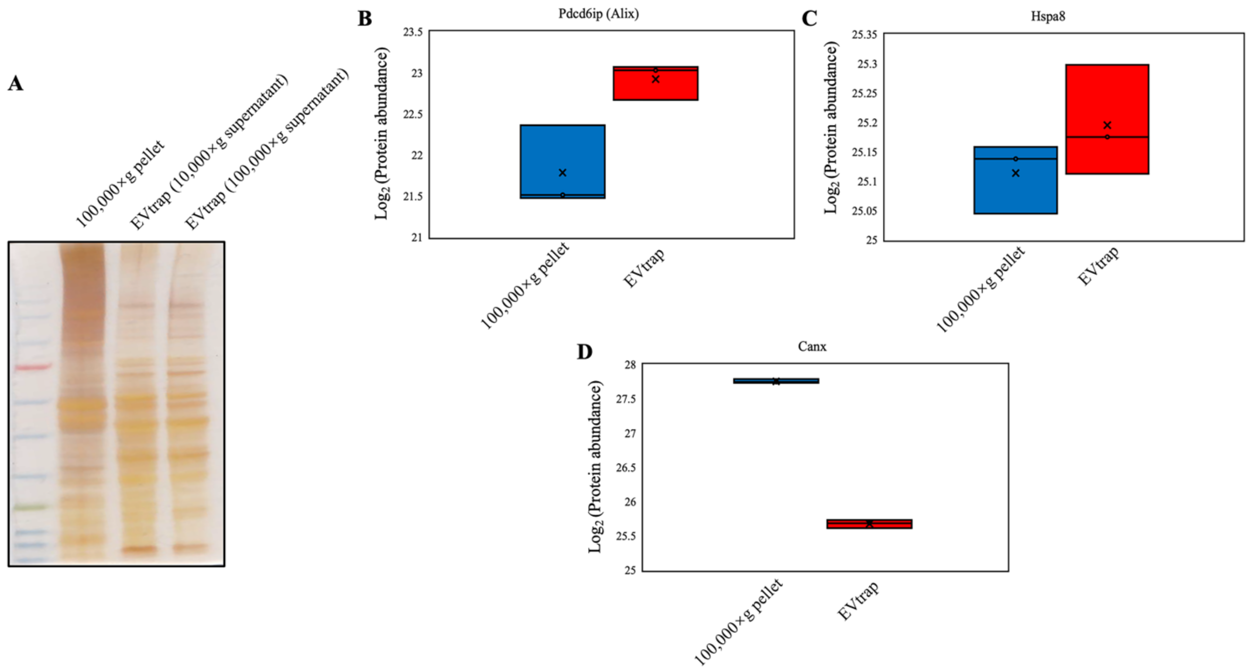

Fig. S2. (*A*) Silver staining demonstrating total protein amounts. LC-MS/MS characterization to support western blotting data of 2 EV markers, (*A*) Pdcd6ip (Alix), (*B*) Hspa8, as well as a contaminant protein, (*C*) Canx. EVtrap was performed on 10,000×g supernatant. Equally 0.5 µg resulting peptides from each method were loaded and technical triplicates were employed.

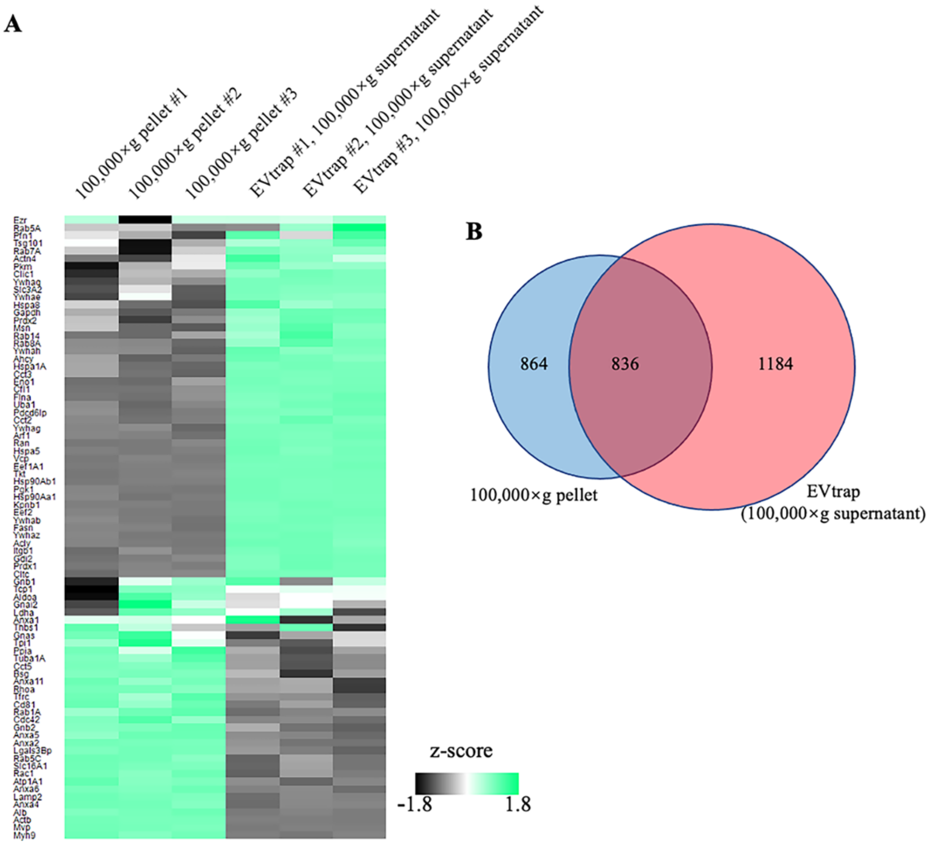

Fig. S3. EVtrap on 100,000×g supernatant to illustrate full recovery of liver tissue EVs. (*A*) Hierarchical clustering analyses of representative EV markers by LC-MS/MS characterization. (*B*) The numbers of identified EV proteins by ultracentrifugation and EVtrap (starting from 100,000×g supernatant). 0.5 µg resulting peptides from each method were loaded and technical triplicates were employed. Selected proteins were identified in at least 2 out of 3 technical replicates.

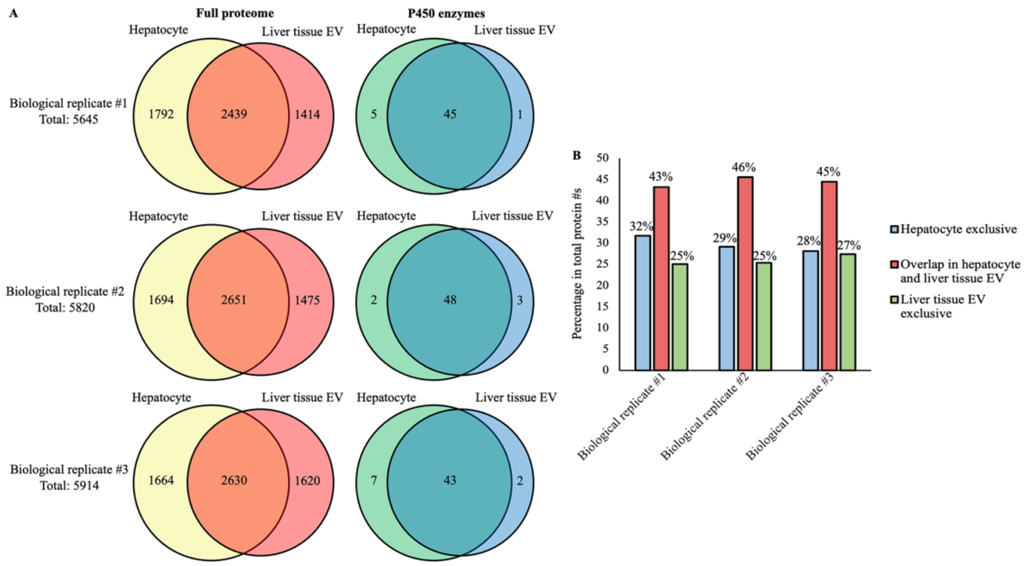

Fig. S4. (*A*) Detailed protein numbers and (*B*) percentages in each hepatocyte and liver tissue EV biological replicate by LC-MS/MS.

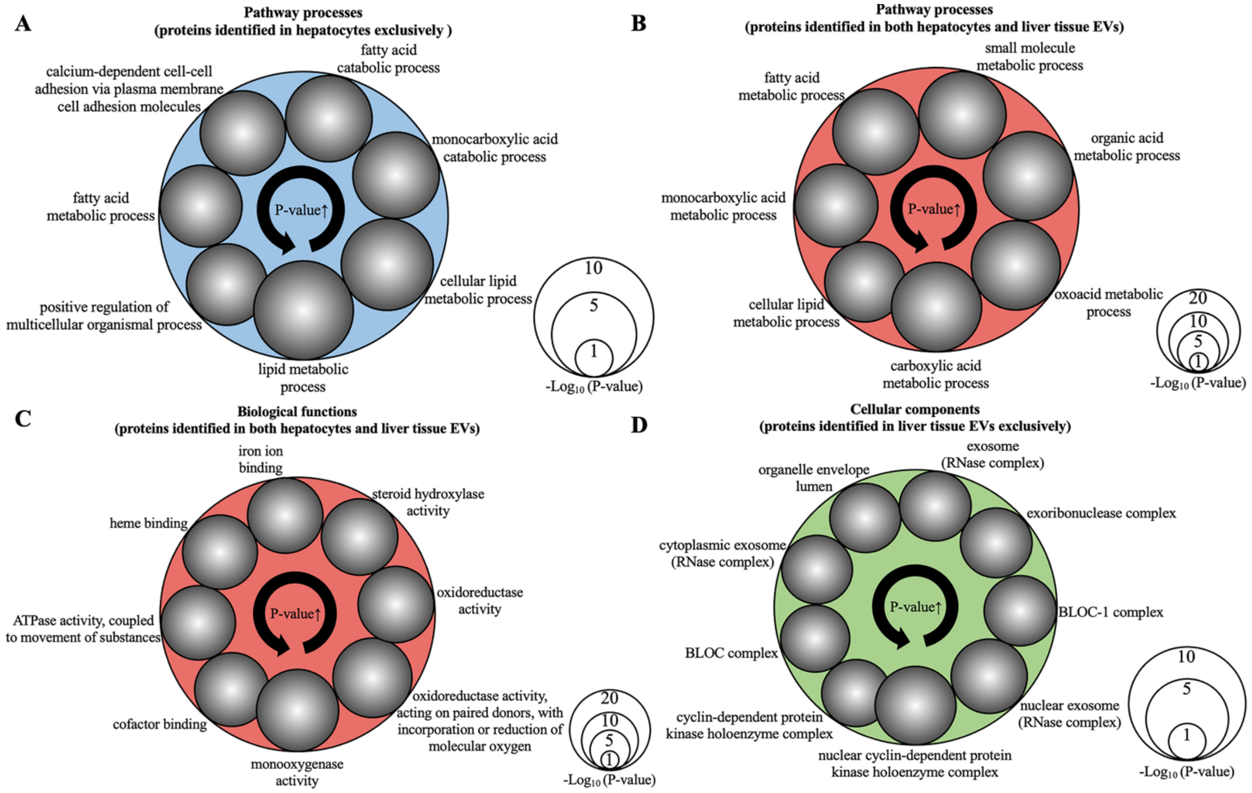

Fig. S5. Classification based on pathway processes, biological functions and cellular components, identified proteins from (*A*) hepatocytes exclusively, (*B-C*) both hepatocytes and liver tissue EVs and (*D*) liver tissue EVs exclusively. Selected proteins in each category were identified in at least 2 out of 3 biological replicates.

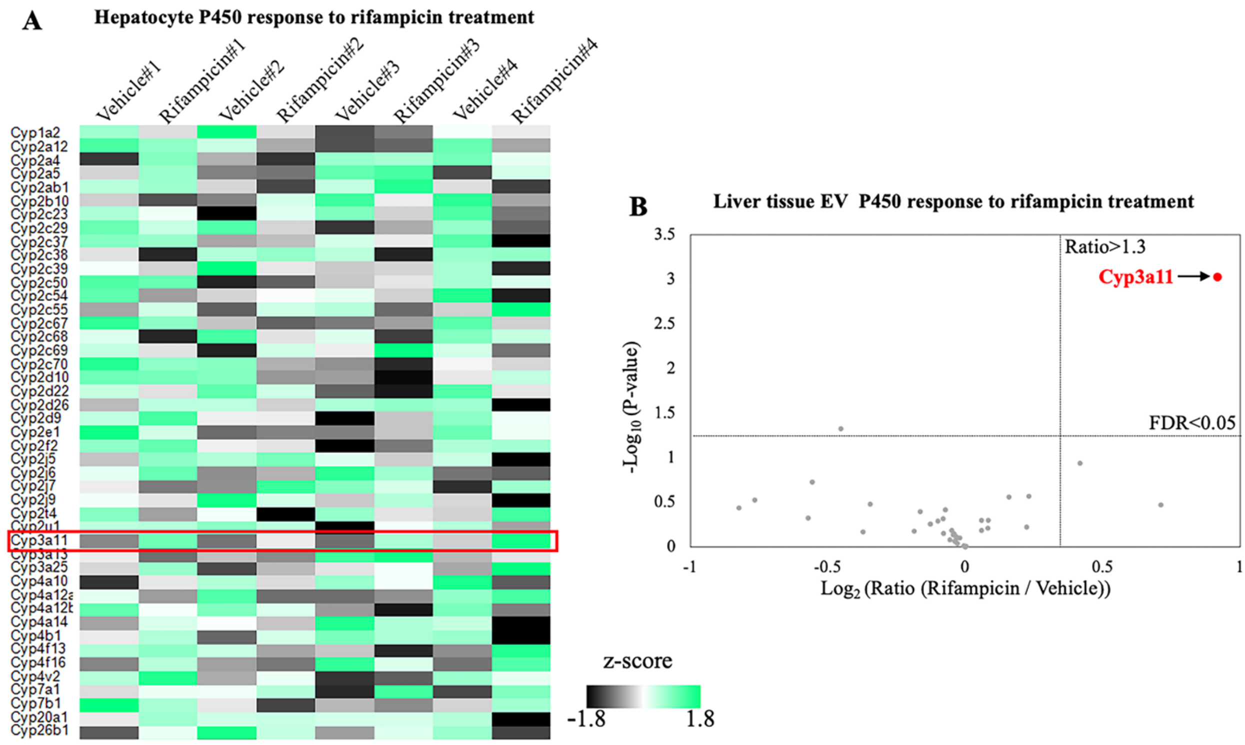

Fig. S6. Heatmap and volcano plotting showing significantly and consistently regulated proteins from hepatocytes (*A*) and liver tissue EVs (*B*), respectively.

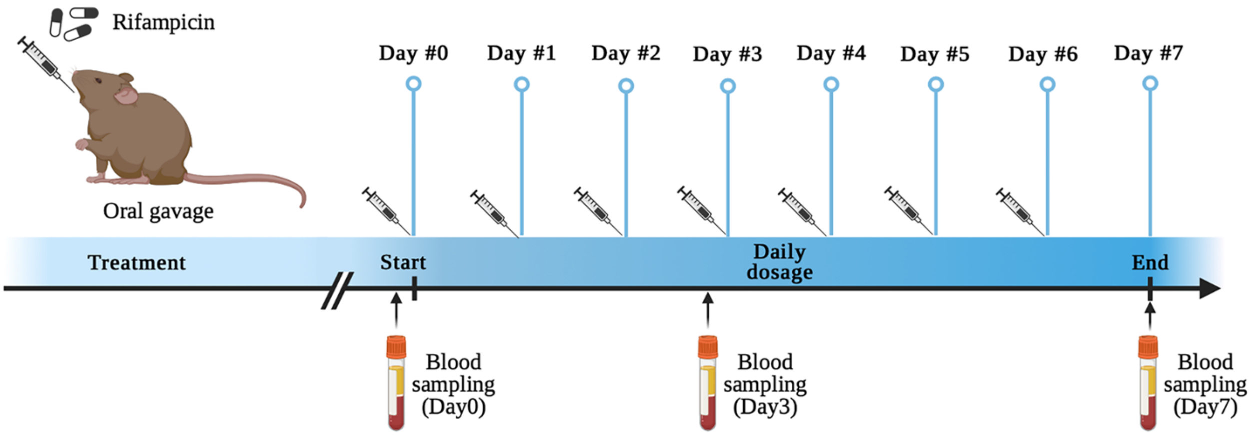

Fig. S7. The strategy for one week rifampicin or dasatinib treatment to collect tail blood.

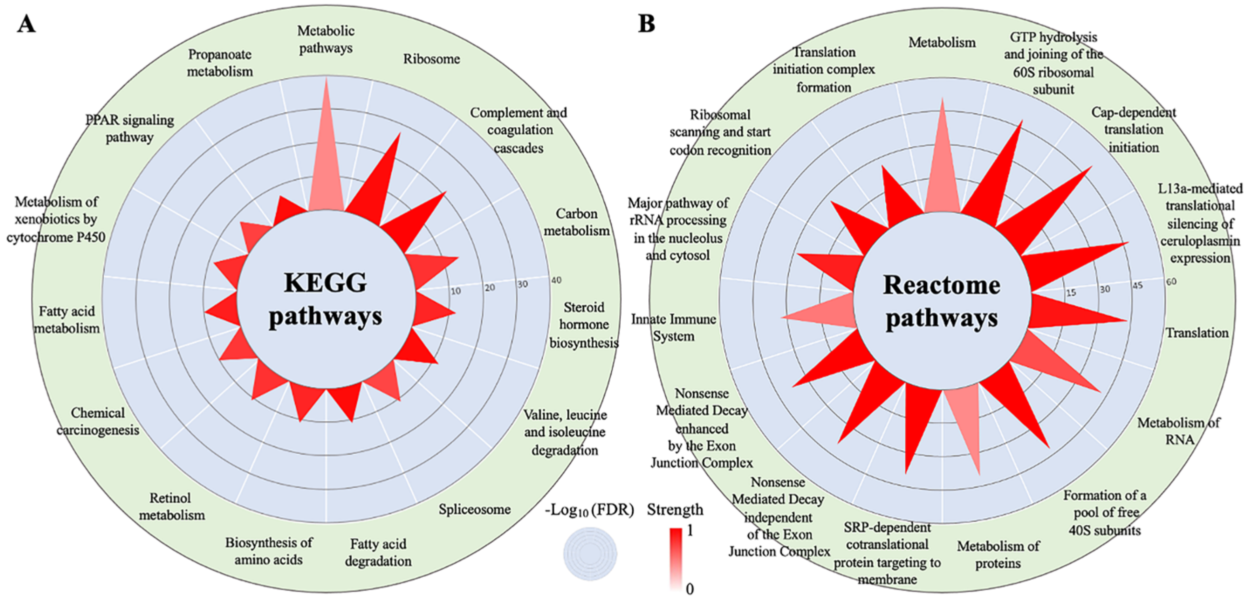

Fig. S8. Classification of identified plasma EV proteins in TMT carrier experiments based on KEGG pathways (*A*) and Reactome pathways (*B*).

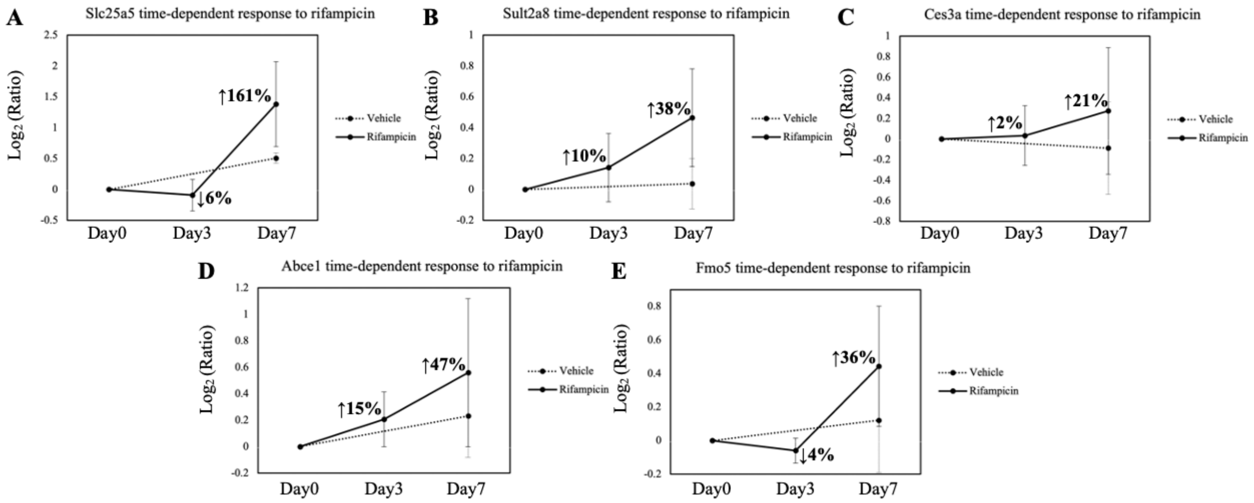

Fig. S9. Rifampicin-stimulation time-dependent response in plasma EVs, in terms of Slc25a5(*A*), Sult2a8 (*B*), Ces3a (*C*), Abce1 (*D*) and Fmo5 (*E*).

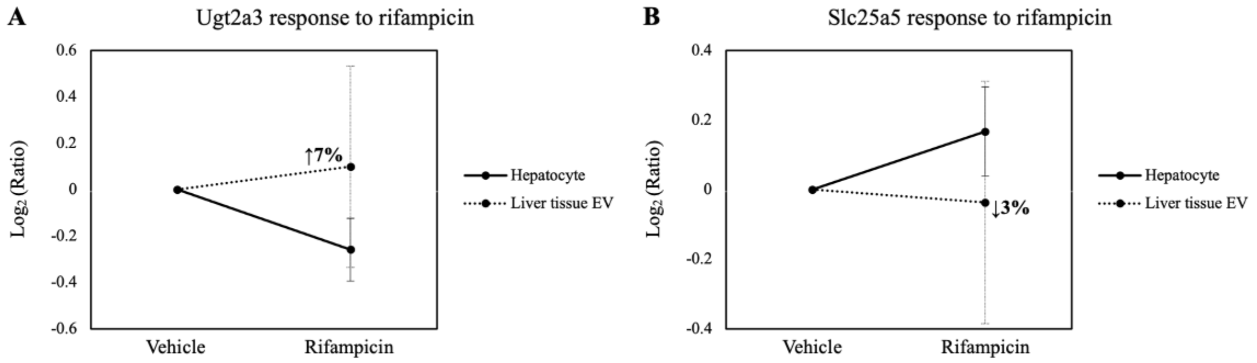

Fig. S10. On day 3, rifampicin-stimulation response in hepatocytes and liver tissue EVs, in terms of Ugt2a3 (*A*) and Slc25a5 (*B*).

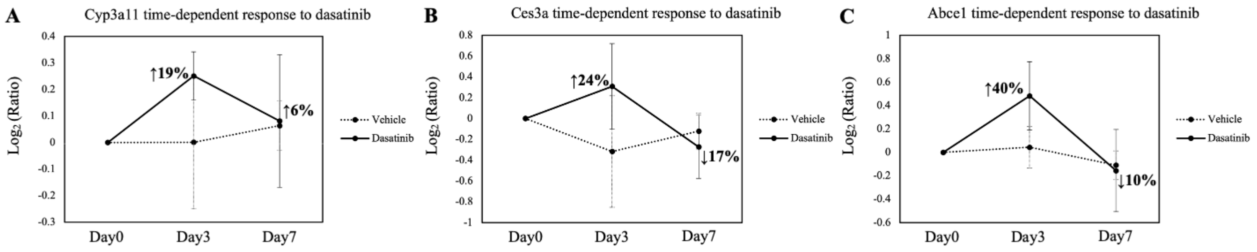

Fig. S11. Dasatinib-stimulation time-dependent response of Cyp3a11 (*A*), Ces3a (*B*) and Abce1 (*C*) in plasma EVs.

Table S1. TRPS data with particle size and concentration information.

Table S2. EV marker abundances in 100,000×g pellet and EVtrap on 10,000×g supernatant, quantified through LC-MS/MS.

Table S3. Common contaminant protein abundances in 100,000×g pellet and EVtrap on 10,000×g supernatant, quantified through LC-MS/MS.

Table S4. Identified proteins in 100,000×g pellet and EVtrap on 10,000×g supernatant through LC-MS/MS.

Table S5. EV marker abundances in 100,000×g pellet and EVtrap on 100,000×g supernatant, quantified through LC-MS/MS.

Table S6. Identified proteins in 100,000×g pellet and EVtrap on 100,000×g supernatant through LC-MS/MS.

Table S7. Identified proteins in hepatocytes and liver tissue EVs.

Table S8. Full proteomic abundances in hepatocyte upon 3-day rifampicin treatment, quantified through LC-MS/MS and normalized by total peptide amount.

Table S9. Full proteomic abundances in liver tissue EV upon 3-day rifampicin treatment, quantified through LC-MS/MS and normalized by EV marker Hspa8.

Table S10. Proteins identified in all three pairs of mouse plasma samples through LC-MS/MS.

Table S11. ADME protein abundances in plasma EV along 7-day rifampicin treatment, quantified through LC-MS/MS and normalized by EV marker Hspa8.

Table S12. ADME protein abundances in plasma EV along 7-day dasatinib treatment, quantified through LC-MS/MS and normalized by EV marker Hspa8.
